# Improving the Accuracy of mmWave Radar for Ethical Patient Monitoring in Mental Health Settings

**DOI:** 10.3390/s24186074

**Published:** 2024-09-19

**Authors:** Colm Dowling, Hadi Larijani, Mike Mannion, Matt Marais, Simon Black

**Affiliations:** 1 School of Computing, Engineering and Built Environment, Glasgow Caledonian University, Glasgow G4 0BA, UK; 2 Safehinge Primera, Glasgow G4 9TH, UK

**Keywords:** patient monitoring, RF sensing, radar, healthcare, suicide, patient safety, multipath reflections, unscented Kalman filter, UKF, person tracking

## Abstract

Monitoring patient safety in high-risk mental health environments is a challenge for clinical staff. There has been a recent increase in the adoption of contactless sensing solutions for remote patient monitoring. mmWave radar is a technology that has high potential in this field due it its low cost and protection of privacy; however, it is prone to multipath reflections and other sources of environmental noise. This paper discusses some of the challenges in mmWave remote sensing applications for patient safety in mental health wards. In line with these challenges, we propose a novel low-data solution to mitigate the impact of multipath reflections and other sources of noise in mmWave sensing. Our solution uses an unscented Kalman filter for target tracking over time and analyses features of movement to determine whether targets are human or not. We chose a commercial off-the-shelf radar and compared the accuracy and reliability of sensor measurements before and after applying our solution. Our results show a marked decrease in false positives and false negatives during human target tracking, as well as an improvement in spatial location detection in a two-dimensional space. These improvements demonstrate how a simple low-data solution can improve existing mmWave sensors, making them more suitable for patient safety solutions in high-risk environments.

## 1. Introduction

### 1.1. Background

Monitoring patient safety is a key challenge for nursing staff in mental health wards. Patients in these environments have a high risk of suicidal thoughts and behaviours, while some may also pose a risk of self-harm or harm to others. The UK National Confidential Inquiry into Suicide and Safety in Mental Health (NCISH) reports that between 2010 and 2020, there were 18,403 suicide deaths in the UK by patients in contact with mental health services [1]. Incidents of self-harm are also concerning, with 102,733 incidents reported by NHS England in the year 2023 [2]. The current approaches to monitoring patient safety involve the use of specialised risk assessments in conjunction with regular physical observations by nursing staff; however, this approach has its limitations, as follows:

1. It places a high burden on staff time, which could be used to support patient recovery.
2. It provides low observability rates—if a patient is being observed for 30 s every 15 min, this is only 3% coverage over a day.
3. Repeated physical observations can be disruptive to sleep and patient privacy as they require nursing staff to physical enter the room to verify that a person is alive. Sleep is an essential component of recovery, so protecting sleep should be seen as a priority.
4. Observation logs by staff members may be prone to bias and error.

Remote sensing solutions with real-time technology for monitoring patient behaviours e.g., wearable sensors, camera-based systems, and radar-systems are already available [3,4,5,6,7,8]. They have high value to inpatient mental health settings as they can help to identify several metrics that indicate if a person is at risk. Knowing the real-time location enables staff to respond to changes which may indicate a period of high risk (e.g., if a person leaves their bed during the night, if a person is on the floor or in proximity to a ligature point). These data can be aggregated to infer indicators of wellbeing, such as activity, restlessness, or sleep hygiene. However, when developing and deploying these technologies, ethical considerations must also be given to their impact on patient privacy and dignity, wellbeing and recovery, and staff–patient relationships. Each technology solution has its own strengths and weaknesses.

Wearable sensors [9,10] equipped with unique identifiers offer a direct method of monitoring individual movements and can provide real-time location data. However, they raise issues around energy consumption, comfort, privacy, and user compliance. Camera-based systems utilise computer vision algorithms to detect, track, and identify targets within a space and remove the need for patients to carry devices or tags, but they raise serious concerns around privacy, consent, data security, and impact on patient wellbeing [11,12,13,14,15]. Furthermore, they are prone to interference by light, dust, and physical obstructions. Empirical evidence suggests that the negative impacts of video surveillance on patient wellbeing outweigh the benefits [11,16].

mmWave radar is an alternative method of tracking human targets using radio frequency (RF) signals to detect and analyse human movements. mmWave has become a popular choice for remote patient monitoring solutions due to its low cost and protection of privacy, leading to a number of commercial off-the-shelf mmWave solutions for lower-risk care settings. However, there are challenges in adapting these sensors for use in higher-risk settings due to their vulnerability to interference from noise sources. This paper discusses the strengths and limitations of mmWave for medium- to high-risk mental health environments. It then proposes a novel low-data solution to improve the reliability of the mmWave detection of human targets using a commercial off-the-shelf sensor.

### 1.2. mmWave Radar Applications in Healthcare Settings

mmWave radar operates in the electromagnetic spectrum between 30 and 300 GHz. It uses short wavelengths of 1–10 mm to achieve high spatial resolution, making it the preferred choice for applications requiring high spatial detection and distance calculation. mmWave solutions do not require wearable tags or identifiable data to determine a person’s location. They are robust to changes in lighting and can penetrate physical obstructions, such as dust, blankets, or plastic, making them more suitable for high-risk environments where sensor housings must be anti-ligature by design.

The RF signals emitted by the sensor are reflected by objects in the environment, allowing the radar to calculate the distance and position based on the time delay and angle of return. Radar performance can be enhanced by multiple-input/multiple-output (MIMO) technology, i.e., multiple transmitters and receiver antennae create a virtual array, enabling better angular resolution and improving the spatial detection. Signal processing techniques, such as beamforming, spatial filtering and multiplexing, have been applied to MIMO configurations to further enhance their reliability [17,18,19].

In the field of healthcare, Alhazmi and colleagues [20] presented a real-time activity monitoring system that can monitor physical activity in the home. They used a PointNet neural network to classify common activities including standing, walking, sitting, lying, and falling with an accuracy of 99.5%. Fall detection is an area of particular interest for mmWave radar and has notable impact on mental health inpatient settings, where low-level ligatures are a significant risk. Several studies have developed real-time fall detection systems using mmWave radar that achieve high accuracy and low false alarm rates. Deep learning methods, such as CNNs [21,22], LSTMs [23], RNNs [24], and ensemble learning [25], have shown particular value in the detection of fall events. Hicheri and colleagues [26] demonstrated the ability of a distributed 2 × 2 MIMO system to monitor gait velocity. Other studies have found that mmWave can identify gait parameters, such as stride length, speed, and cadence, which can provide relevant indicators for diseases such as Parkinson’s.

Other common applications in healthcare environments include vital sign detection and sleep monitoring. Chuang and colleagues [27] presented a 60 GHz vital sign solution that extracts breathing and heart rate signals from V-band signals. Yang and colleagues [28] found that 60G Hz radar could locate a human subject with 98.4% accuracy, while achieving a mean error of 0.43 bpm for respiration and 2.15 bpm for heartrate. Ahmad and colleagues [29] showed that vital signs could be extracted from multiple targets using a single mmWave sensor operating at 77–81GHz. Wang and colleagues [30] reviewed applications for sleep monitoring and found that beyond the use of vital signs as indicators for sleep disorders, mmWave sensors could estimate sleep posture, classify different sleep stages, and detect obstructive sleep apnoea.

In practice, mmWave solutions can be problematic in some settings. As the reflected signal does not inherently contain the target identity, target-tracking algorithms are required to assign and track target IDs over time. This is made more challenging by the fact that metals and moisture can reflect or attenuate signals, leading to a phenomenon known as multipath reflections when the RF signals bounce off various surfaces before returning to the radar [31,32,33]. As the paths can vary in length, and the angle and time of arrival may be different and/or overlap, the effects can be as follows:

- Ghost targets (false positives), which can cause staff to conduct unnecessary checks, leading to alert fatigue and distrust in the system.
- Increased background noise (false negatives), which can cause staff to believe a room is unoccupied when it is not.
- Reduced resolution due to the overlapping of return signals.

### 1.3. Target Tracking and Multipath Reflections

Several approaches have been adopted to mitigate the impact of multipath reflections in RF target tracking. Some of these, such as ray-tracing [34,35] and reflection mapping [33], involve aligning the multipath ghosts to the locations of real targets. The use of distributed compressed sensing (DCS) has been shown to lead to significant accuracy improvements and better performance noisy environments [36]. Other methods utilise machine learning (ML) methods on the radar signals to discern the presence of real targets from false targets [37,38,39]. These methods can offer significant improvements in target tracking accuracy but are often computationally intensive or require high volumes of data and considerable time to train, making them impractical to implement with real-time data on embedded systems. Additionally, models that are trained on a specific antennae configuration may not generalise well to other sensors. There is a need for low-data sensor-agnostic solutions that can post-process target location data from RF-based location sensors to discern human from non-human targets. Such solutions must address two concurrent challenges:

(A) Tracking consistent target IDs from locational data across successive time points (as sensors inherently lack the capability to distinguish between targets from one moment to the next);
(B) Discerning real targets from false targets based on their movement properties over time.

Several approaches have been proposed for tracking the locations of human targets over time using radar systems. While tracking consistent target IDs from sensor data, the challenges include data noise, occlusions, and variable target dynamics. There is often a trade-off between a method’s computational complexity and robustness to dynamic environments. Kalman filters provide a means of estimating the state of the target by integrating measurement data with a linear dynamic model. They are efficient, well-suited for real-time applications, and can be combined with data association techniques, such as the nearest neighbour algorithm. They have low computational overhead and offer ease of implementation, but struggle with the inherent non-linearity in human behaviour [40]. 

Several alternative approaches have been proposed to manage the inherent non-linearity of human motion. Particle filters [41,42] use a set of discrete particles to represent the probability distribution of target states. They are highly effective in modelling diverse motion patterns, but they often struggle with higher numbers of targets and are computationally intensive. Joint probabilistic data association filters [43,44] can handle multiple target tracking scenarios and are effective in non-linear data assignment; however, they also have a high computational cost. Deep learning approaches have become increasingly common [45] and are all highly adaptable to non-linear movement patterns, but they require extensive training, and there is a risk of overfitting to particular environments. They also come with a high computational cost.

Recent studies have looked into methods of extending the traditional Kalman approach to handle non-linear data. The unscented Kalman filter (UKF) has been gaining popularity for its ability to accurately approximate targets with low computational costs, even in situations of extreme nonlinearity [46]. In contrast to the traditional Kalman filter, which assumes that both the process and observation models are linear, the unscented Kalman accommodates nonlinear relationships by specifying a set of deterministically chosen sigma points. It uses these points to compute the state estimation by propagating them through nonlinear functions and using their transformed positions to update the state and certainty. While it has been heavily utilised in aerial tracking and autonomous vehicles, there is less evidence of its value in human tracking within indoor closed environments.

The rest of this paper describes a simple low-data solution for improving the reliability of mmWave target detection. We implemented a UKF to track the location of targets over time, and then used motion pattern analysis (MPA) to identify which targets were true based on their patterns of movement. The focus of this research was using a combination of UKF and MPA to improve the spatial resolution of the sensor and to reduce the numbers of false positives (FP) and false negatives (FN).

## 2. Materials and Methods

**Figure 1:**
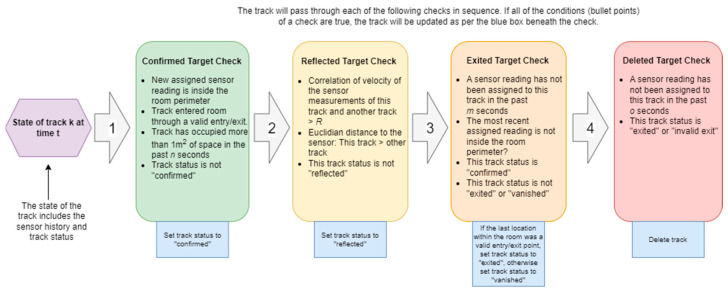
Motion pattern analysis check process.

To validate our solution, we created a simulated patient bedroom environment and had a human participant walk around the room while a mmWave sensor tracked their movement. We compared the initial output of the sensor against the output after applying the UKF and MPA (Figure 1) to identify whether we could successfully improve the accuracy of detections.

### 2.1. mmWave Radar 

We chose a commercial off-the-shelf mmWave 60 GHz MIMO radar to identify the locations of human targets in a simulated mental health patient bedroom. The sensor chosen was the Vayyar Care Type C device running version 37045 of the standard firmware [47]. This sensor was selected for its high performance in elderly care settings [48], a 140° field of view, built-in algorithms for target identification, and compatibility with common wireless communication protocols (HTTP and MQTT).

The sensor operates by transmitting and receiving mmWave signals from 46 antennae within a defined indoor space. The mmWave signals are reflected by different targets in the environment, providing a 3D point-cloud of the target locations. The sensor includes built-in algorithms for environmental noise mapping and identifying human targets from the point-cloud data. The sensor output consists of a list of X, Y, and Z co-ordinates at each time point and does not include raw RF signal data. The data transmission rate was set at 5 Hz, as this was the maximum packet rate of the sensor. Data packets containing target information were sent via MQTT to a Rock4 SE single-board computer over a local network, where they were then written to a log file. 

### 2.2. Experimental Setup

**Figure 2:**
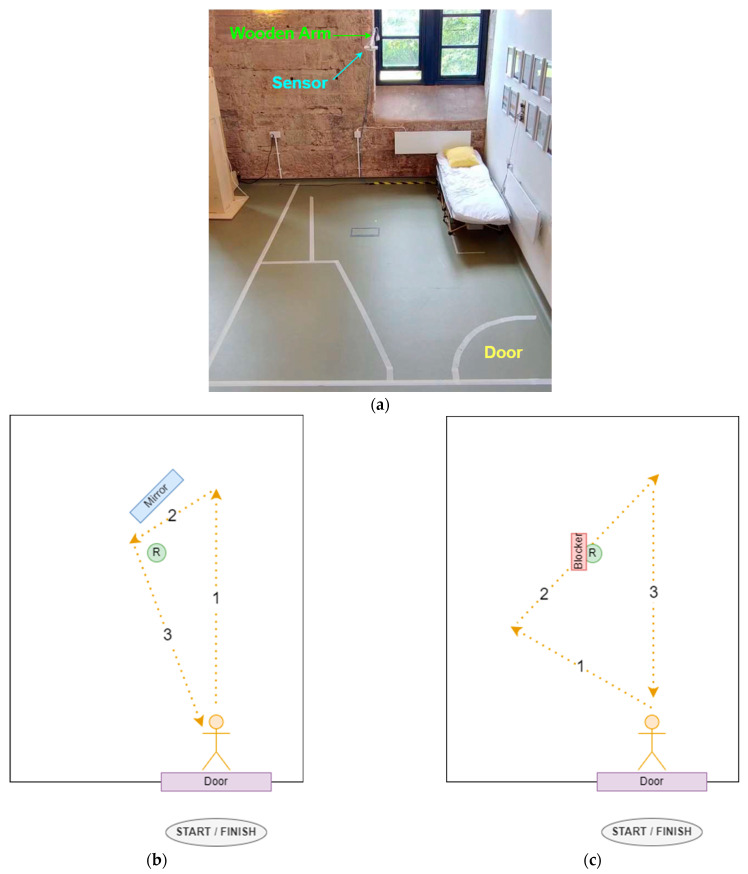
Experimental setup: (**a**) simulated patient bedroom layout, with a sensor mounted on a wooden arm in the centre of the room; (**b**) Experiment 1 setup and target trajectory; (**c**) Experiment 2 setup and target trajectory.

To test the base performance of the radar system, two experiments were conducted in a 2.7 m by 3.8 m simulated mental-health patient bedroom (see Figure 2a). The radar sensor was positioned 2.1 m above the floor on a wooden arm mounted to one of the walls. In each experiment, a single human participant walked around a room in a fixed trajectory. Obstructive objects were placed close to the radar in order to (negatively impact) the sensing accuracy. 

Experiment 1 involved placing a mirror next to the radar sensor in order to reflect the mmWave signals (Figure 2b). This increased the likelihood of false targets being detected by the sensor. For Experiment 2, a sheet of metal foil was placed along one side of the radar sensor in order to create a blind spot in the sensor area (Figure 2c). This increased the likelihood of the sensor failing to detect a genuine target. Each experiment consisted of 10 trials where a person followed a predetermined trajectory around the room (however, one trial from Experiment 2 was excluded due to an error in the video recording). The trajectories were chosen to maximise the impact of the obstructive objects on the sensor recording.

### 2.3. Data Collection

During each trial, a person entered the room through a doorway and walked along a trajectory before exiting the room through the same location. Figure 2b shows the walking trajectory for each experimental setup. The yellow lines numbered 1–3 indicate the direction that a person walked around the room during each trial. All trials involved a single person, and walking speeds varied between trials. The walking speed was intentionally not controlled in order to emulate the natural differences in walking speeds in a typical ward environment. Each trial began when a participant entered the room and ended when they exited the room. The shortest trial was 14 s, and the longest was 31 s due to the varying walking speeds and differences in trajectory length between Experiments 1 and 2. The trials were conducted over the course of a single day.

During each trial, the mmWave sensor recorded live data, which were timestamped and stored on a Rock4 SE single-board computer. In order to generate a ground truth for the locations of the targets, a OnePlus 12 mobile phone camera was used to collect a video feed. To identify the true target locations, the video footage was viewed, and the target locations were recorded into a spreadsheet rounded to the nearest 0.25 m. Gridlines were marked on the floor every 0.5 m to simplify the target annotation.

### 2.4. Data Preprocessing

To compare the initial performance of the sensor output, the sensor data at each time point were compared against the ground truth, and each time point was scored as follows:

- **True positive:** If there was only 1 target detected by the sensor;
- **False positive:** If there was more than 1 target detected by the sensor;
- **False negative:** If there was less than 1 target detected by the sensor.

This scoring was based on the fact that as there was only one subject in all trials, the sensor output should only report one target. We also calculated a distance accuracy metric based on the Euclidian distance between the ground truth and its nearest sensor target. The trial performance metrics (TP, FP, FN, and distance accuracy) were calculated before and after applying the UKF and MPA. Table 1 shows an example of the trial outputs.

### 2.5. Target Tracking and Classification Using UKF and MPA

**Figure 3:**
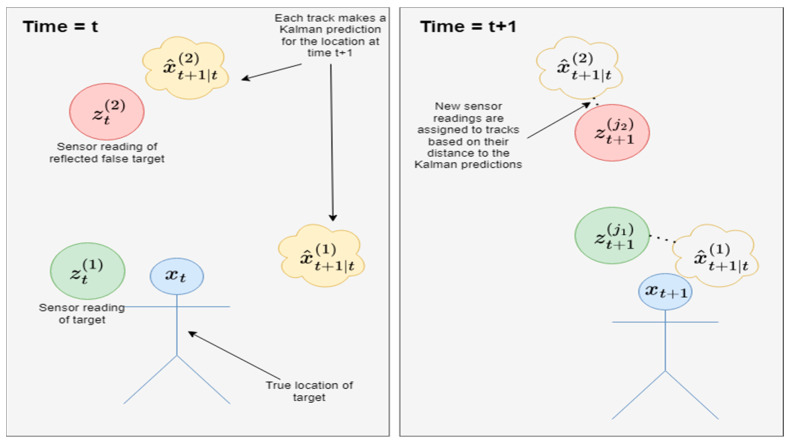
Kalman prediction and target assignment.

To improve the target detection and tracking, we fed the log file containing the sensor output data into a pipeline consisting of several steps. The first step was to identify target continuity over time. In order to assign continuous target IDs to each sensor reading, an unscented Kalman filter (UKF) was used to make a prediction at each time point *t* for the location of the target at *t* + 1 (Figure 3). The UKF was chosen for its high performance on non-linear data. A constant acceleration model was used for the state transition function. Once the predictions were made, the Kuhn–Munkres algorithm was used to assign the detected targets to tracks. A cost matrix was generated using the Euclidian distance between the sensor reading at t + 1 and the Kalman prediction. A penalty was applied to costs above 300 cm, as it would be unusual for a human target to move at such a high speed. A linear sum assignment step returned the optimal assignment of new sensor targets to existing tracks. At the end of this step, if there were any unassigned targets, a new track ID was created for each, comprising the current timestamp and a unique 3-digit identifier.

The cost function and optimal assignment can be expressed as follows, where ${\hat{x}}_{t+1|t}^{\left(i\right)}$ is the *i*-th Kalman prediction at time *t* + 1, and $z_{t+1}^{\left(j\right)}$ is the *j*-th new sensor location at time *t* + 1:

(i) Euclidian distance $d_{ij}=||{\hat{x}}_{t+1|t}^{\left(i\right)}-z_{t+1}^{\left(j\right)}||$
(ii) Cost function $c_{ij}=\left\{\begin{array}{c} d_{ij}\;if\;d_{ij}\leq \;300\\ d_{ij}+10000\;if\;d_{ij}>300 \end{array}\right.$
(iii) Optimal assignment ${min}_{{\sigma \in S}_{n}}\sum_{i=1}^{n}c_{i,\sigma (i)}$

After each target was assigned to a track (the track consisted of an array of sensor locations at each time point and their associated Kalman predictions), the status of the track was then updated. Determining the status of the track involved motion pattern analysis (MPA) of the array of sensor locations assigned to that track. We identified several patterns of movement that are common to human targets. We then mapped this set of patterns of behaviours of real targets to a set of measurements that we validated each track against (Table 2).

In addition to distinguishing between real and false targets, the monitoring system needed to identify when the sensor had failed to identify a target (i.e., the target is missing) and when a target legitimately left the room. In order to carry this out, we used the entry/exit point used in Measurement #1 of Table 2 to determine whether a target had left the room.

From this, we were able to assign a status to each track:

- **Ghost target**—All new targets begin as a ghost target.
- **Confirmed target**—When a ghost target meets Measurements 1 and 2 of Table 2, it becomes a confirmed target.
- **Reflected target**—When a target has a high velocity correlation with another target and is further from the sensor than that target, it becomes a reflected target.
- **Exited target**—When a target has not been assigned a new sensor reading for X seconds, and its last assigned sensor reading was within an “entry/exit point”, it becomes an exited target.
- **Vanished target**—When a target has not been assigned a new sensor reading for X seconds, and its last assigned sensor reading was not within an “entry/exit point”, it becomes a vanished target.

In order to prevent memory creep, we added a timeout for exited and vanished tracks. After 10 s, if no new sensor readings were identified, the target tracks were removed from local memory.

At each time point, the output of our pipeline was a set of target tracks, which contained the X, Y, Z sensor target, the Kalman prediction, and the track status at that time point. This was saved to a log file. To compare the performance after the UKF and MPA were applied, we extracted only the targets where the track status was “confirmed” and compared them against the original sensor output.

## 3. Results

**Figure 4:**
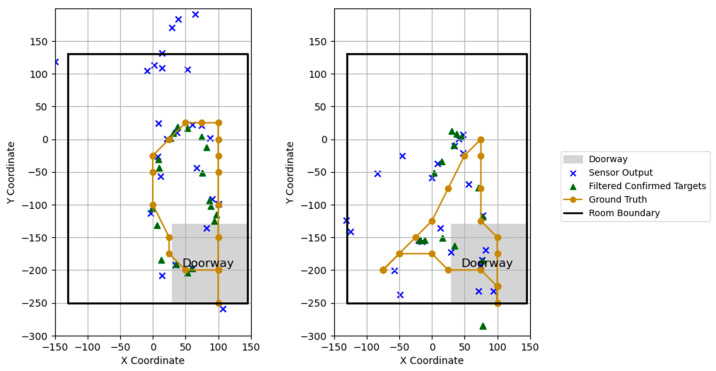
Location data for 2 trials consisting of radar sensor output, filtered targets, and ground truth.

**Figure 5:**
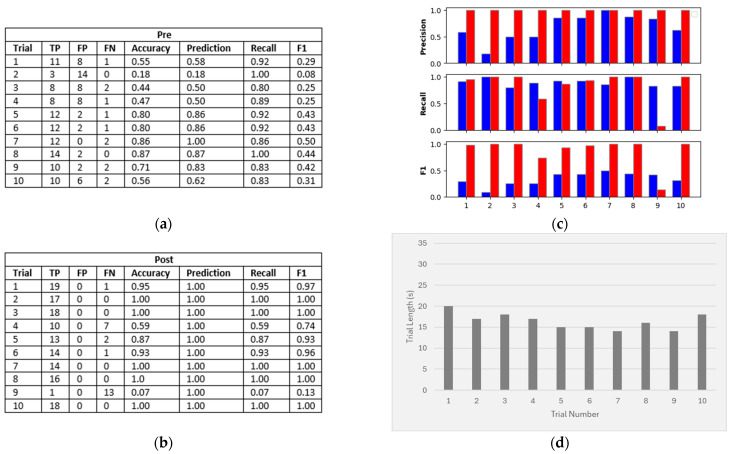
Experiment 1: (**a**) Results of each trial before target tracking and MPA were applied (pre). (**b**) Results of each trial after applying target tracking and MPA (post). (**c**) Comparison of precision, recall, and F1 for each trial (pre (blue) and post (red)). (**d**) Trial durations.

**Figure 6:**
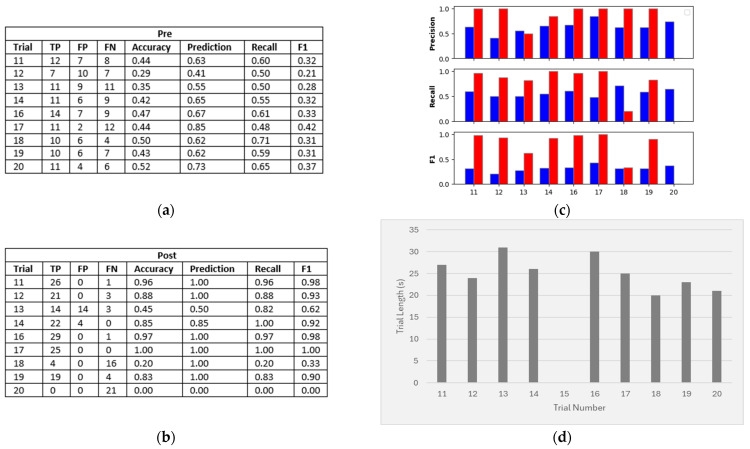
Experiment 2: (**a**) Results of each trial before target tracking and MPA were applied (pre). (**b**) Results of each trial after applying target tracking and MPA (post). (**c**) Comparison of precision, recall, and F1 for each trial (pre (blue) and post (red)). (**d**) Trial durations (note that trial 15 was excluded due to an error in the video recording).

In order to compare the performance of our pipeline output against the initial sensor output data, we determined the (pre) number of true positives, false positives, and false negatives on each trial. We used this to generate a sensitivity, specificity, and F1 score for each trial. We then ran target tracking and MPA on the data and removed any targets that did not have the “confirmed” status. Figure 4 shows a sample trial from each experiment, highlighting the difference before and after tracking and MPA. We then determined the (post) number of true positives, false positives, and false negatives of the output (Figure 5 and Figure 6). Due to the variable lengths of the trials, the combined total of true positives (TPs), false positives (FPs), and false negatives (FNs) differed across the trials. However, for each individual trial, the sum of the TPs, FPs, and FNs was equal to the length of that trial (Figure 5 and Figure 6).

### 3.1. Experiment 1

Experiment 1 consisted of 10 trials and involved placing a mirror next to the radar sensor while a person walked a set path across a room (Figure 2b). The aim of this experiment was to see whether the reflected targets generated by the mirror could be removed by target tracking and MPA. This experiment did not consider the accuracy of the target location, only whether the correct number of targets were detected. Figure 5 shows the results for each trial in Experiment 1. Before target tracking and MPA was applied, the results show an average prediction across the trials of 68%, average recall across the trials of 90%, and an average F1 score across the trials of 34%. After applying target tracking and MPA, the average prediction accuracy across the trials increased to 100%, and the average F1 score across the trials increased to 87%. This suggests that MPA was able to successfully identify false targets generated by reflections.

Despite showing a mean decrease in recall (85%) after tracking and MPA, recall improved in 7 out of the 10 trials. However, there was a large decrease in recall in trial 9 (see Figure 5c), which negatively impacted the overall mean. This was due to a previously detected reflected target disappearing at the same time that a new true target appeared. This means that the new true target was assigned to the reflected target’s track and was, therefore, mistakenly classified as a reflected target.

### 3.2. Experiment 2

Experiment 2 consisted of nine trials (trial 15 was excluded due to an error in the video recording) and involved placing a thick sheet of metal foil over one side of the sensor to create a blind spot. A target then walked a set path behind the blind spot before returning into the sensor region (Figure 2c). The aim of this experiment was to see whether target tracking and MPA successfully tracked the target identity, even when the target was not detected by the sensor. This experiment did not consider the accuracy of the target location, only whether the correct number of targets was detected. Figure 6 shows the results for each trial in Experiment 2. Before target tracking and MPA were applied, the results show an average prediction across the trials of 64%, average recall across the trials of 58%, and an average F1 score across the trials of 32%. After applying target tracking and MPA, the average prediction accuracy across the trials increased to 82%, the average recall across the trials increased to 74%, and the average F1 score across the trials increased to 74%. This indicates that the impact of missing targets (FNs) was minimised by our tracking and MPA.

Two trials (trial 18 and trial 20) showed worse performance than the others. In both cases, this occurred due to a true target with an extremely low velocity having a high correlation with a stationary false target, which led to the true target being incorrectly classified as a reflection.

### 3.3. Experiment 3

The third experiment looked at whether the Kalman prediction yielded better target detection accuracy than the initial sensor output data. To carry this out, we calculated D1 as the Euclidian distance at each time point between the ground truth and the nearest sensor target, and D2 as the Euclidian distance at each time point between the ground truth and the Kalman prediction. The rounding of the ground truth to the nearest 0.25 m indicates that there is inherent imprecision in the ground truth data, possibly leading to an overestimation or underestimation of true errors. Additionally, it would be unusual for distances to be normally distributed due to their bounded nature (i.e., distances are always positive). To correct for these factors, we chose a non-parametric test for comparing the differences in distance in the pre (D1) and post (D2) conditions.

The descriptive statistics indicate that there was a difference between D1 (mean = 44.20, SD = 31.08) and D2 (mean = 39.86, SD = 26.60). The results of a Shapiro–Wilks test support our assumptions that the differences were not normally distributed (*p* < 0.001). In assessing the effectiveness of the Kalman filter on reducing the distance errors, a Wilcoxon signed-rank test was conducted. The analysis revealed a statistically significant decrease in distance error after applying the Kalman filter (W = 19920, df = 317, *p* < 0.001). This indicates that the UKF led to a small but significant improvement in tracking accuracy.

## 4. Discussion

The aim of this paper was to use a simple low-data solution to improve the target tracking accuracy of a commercial off-the-shelf RF sensor while reducing the number of FPs and FNs. The findings suggest that the UKF predicted the true location of the target with better accuracy than the initial sensor output. Furthermore, the results found that applying motion pattern analysis to classify true targets dramatically reduced the number of FPs and FNs detected by the sensor. The use of the UKF has been extensively explored in other areas, such as aerial tracking and autonomous vehicles, but its use in human tracking has been less explored to date.

This approach has several advantages over existing methods for multipath mitigation/exploitation. It is cheap and straightforward to set up and has a low computational overhead. In contrast to deep learning methods or other supervised learning approaches, this does not require extensive training to achieve high performance. It has comparatively few parameters that need to be optimised and can be run on a 4GB single-board computer, which provides advantages in healthcare settings, where the physical limitations of buildings often make it challenging to install large pieces of hardware. The use of mmWave provides further advantages for these settings due to its smaller packet size. mmWave sensor packets are typically under a few kilobytes, whereas RGB-D camera packets can often be hundreds of megabytes in size. This is significant for healthcare environments, where bandwidth is often limited and delays in messaging can have serious consequences.

In addition, this method does not rely on a particular antennae configuration or even particular sensor, as the input data are just a series of X, Y, and Z co-ordinates. This system could, therefore, provide many benefits for healthcare settings. Our findings show that non-visual sensors can reliably detect humans with a high degree of accuracy. This can help support staff know where a patient is without compromising their right to privacy and dignity. By identifying whether a patient is out of bed at night, or whether they are in close proximity to a ligature point, it is possible to raise an alert for staff to investigate. This can lead to a reduced number of serious incidents and improve the safety of these environments.

There are several limitations to our approach that should be considered. Perhaps the most significant is that our experiments only used a single participant. Repeating the experiment with multiple participants would establish how well the sensor can distinguish between two real targets. Another limitation is that our experiment only used a single approach for target tracking. There would be a benefit to compare the performance of the UKF with other methods (e.g., traditional and extended Kalman filters) to identify whether similar improvements could be achieved with even less computational demand. Additionally, our Kuhn–Munkres algorithm used Euclidian distance as a cost function. As a result, it essentially operated as a global nearest neighbour. While this has been demonstrated as a simple and effective method of assigning targets over time, it may be possible to achieve better results by modifying the cost function to include additional target aspects, such as velocity, acceleration, or variability in movement.

While our MPA model improved the sensor performance in most trials, it performed poorly in cases where one target disappeared at the same time as and a new person entered the room. While we set a penalty on the cost function to limit targets from “jumping” long distances, this did not account for cases where there was the same number of targets at t-1 and t. This could be mitigated by applying a hard limit on the distance that targets can be assigned and creating a new track if the distance limit is exceeded. We also found that when targets had a low velocity, the model mistakenly classified them as reflections. This was most likely due to the fact that still targets have lower variability in velocity and, therefore, a lower signal-to-noise ratio (SNR). This means that small synchronous movements in any direction can lead to a high correlation coefficient and a misclassification. This could be mitigated in the future by requiring reflected targets to have a minimum mean velocity over a period. Another limitation of our approach was that we only tracked the target movement in two-dimensional space (e.g., using an X and Y coordinate). However, there is the possibility that reflected targets that follow complex paths may have had their axes distorted. It may be possible to improve the detection of reflected targets by tacking the targets in three-dimensional space. This may also yield improvements in target classification.

Finally, the sensor we chose did not provide raw RF data but only the target co-ordinates of the detected targets. Having access to the underlying data could enable better target tracking by providing additional metrics, such as target amplitude and SNR, which could help to distinguish between real and false targets. Despite these limitations, our findings show promise for the potential of unscented Kalman filtering and motion pattern analysis to improve the detection of human targets with RF sensors. This can aid in the development of better solutions for monitoring patient safety in mental health wards and supporting the delivery of better healthcare.

## 5. Conclusions

This paper proposed a simple low-data solution for improving the reliability of mmWave target detection. This research advances the current literature by showing how commercial off-the-shelf sensors can be adapted for higher-risk settings. Motion pattern analysis removed the majority of reflected and noise targets, leading to higher resilience to false positives and false negatives. This improvement in reliability can support the development of better patient monitoring systems, which can preserve privacy and improve patient safety.

This research also expands on the broader literature of target tracking in indoor environments by showing that unscented Kalman filtering can provide reliable tracking of human targets in noisy indoor settings and improve the accuracy of detection. This has high value for the development of privacy-preserving mmWave solutions for mental healthcare environments, as well as broader applications in security and surveillance systems, smart home and building automation, assisted living, and industrial and workplace safety.

Further work is ultimately needed to explore the challenges in scaling this solution to more complex environments (e.g., where tracking targets across multiple sensors may be required). Additionally, long-term validation in real-world settings is necessary in order to fully understand the system’s performance and reliability. However, the results in our study show that large improvements in target tracking can be achieved without the need for extensive data or computational resources. 

In conclusion, the methods proposed in this study enhance the applicability of mmWave technology for medium- to high-risk mental health settings, supporting the development of more reliable and effective patient monitoring systems that can improve the delivery of care and ultimately save lives.

## Figures and Tables

**Table 1:** Example trial data showing sensor output vs. filtered targets after applying UKF and MPA.

Trial	Time	Ground Truth (x, y)	Sensor Output (x, y)	Score (Pre)	Filtered Confirmed Targets (x, y)	Score (Post)
1	11:39:41	(100, −250)	[]	FN	[]	FN
1	11:39:42	(100, −200)	[(82, −136)]	TP	[(59, −197)]	TP
1	11:39:43	(100, −150)	[(101, −99)]	TP	[(94, −125)]	TP
1	11:39:44	(100, −100)	[(101, −99)]	TP	[(97, −115)]	TP
1	11:39:45	(100, −50)	[(91, −91)]	TP	[(89, −102)]	TP
1	11:39:46	(100, −25)	[(67, −44)]	TP	[(86, −94)]	TP
1	11:39:47	(100, 0)	[(88, 2)]	TP	[(76, −51)]	TP
1	11:39:48	(100, 25)	[(74, 21)]	TP	[(82, −12)]	TP
1	11:39:49	(75, 25)	[(61, 22)]	TP	[(74, 4)]	TP
1	11:39:50	(50, 25)	[(9, 24), (2, 113)]	FP	[(53, 17)]	TP
1	11:39:51	(25, 0)	[(37, 10), (−9, 105)]	FP	[(38, 19)]	TP
1	11:39:52	(0, −25)	[(22, 1), (53, 107)]	FP	[(31, 9)]	TP
1	11:39:53	(0, −25)	[(7, −26), (14, 109)]	FP	[(27, 2)]	TP
1	11:39:54	(0, −50)	[(12, −57), (14, 132)]	FP	[(9, −31)]	TP
1	11:39:55	(0, −100)	[(−3, −113), (65, 191)]	FP	[(10, −44)]	TP
1	11:39:56	(25, −150)	[(−150, 118), (39, 184)]	FP	[(−1, −106)]	TP
1	11:39:57	(25, −175)	[(14, −208), (29, 171)]	FP	[(6, −132)]	TP
1	11:39:58	(50, −200)	[(35, −192)]	TP	[(13, −184)]	TP
1	11:39:59	(100, −200)	[(61, −197)]	TP	[(36, −191)]	TP
1	11:40:00	(100, −200)	[(107, −259)]	TP	[(53, −204)]	TP

**Table 2:** Movement patterns of real and false targets were mapped to specific measurements. These measurements were used to determine whether a target’s location history was indicative of a particular movement pattern.

	Movement Pattern	Measurement
1	Real targets first appear at a valid entry point (e.g., a doorway).	Origin at a valid “entry/exit point”.
2	Real targets do not vanish from the centre of the room. Real targets will always exit through a valid exit point (e.g., a doorway).	Exit at a valid “entry/exit point”.
3	False targets created by inanimate objects generally remain within a fixed location (e.g., doors, fans).	Sensor data history of X, Y targets have a range of >1 m^2^ over past 2 s.
4	Reflected targets should have a high correlation of velocity with at least one other target.	Pearson correlation co-efficient of velocity of X, Y sensor readings is >0.8 over the past 2 s.
5	Reflected targets should always be further from the sensor than their true counterparts (law of reflection and Pythagorean theorem).	Distance of true target is < distance of reflected target.

## Data Availability

The original data presented in the study are openly available in FigShare at https://doi.org/10.6084/m9.figshare.26163415 (accessed on 26 August 2024).
